# A Case Report of Surgical Emphysema, Vocal Cord Palsy, and Pneumomediastinum Following Fishhook and Blunt Neck Trauma

**DOI:** 10.7759/cureus.114436

**Published:** 2026-08-12

**Authors:** Parvez Mohiuddin Dar, Ammar Al-Hassani, Sajid Atique, Murad Alahmad, Ruben Peralta, Ayman El-Menyar, Sandro Rizoli, Hassan Al-Thani

**Affiliations:** 1 Trauma Surgery, Hamad General Hospital, Hamad Medical Corporation, Doha, QAT; 2 Surgery, Universidad Nacional Pedro Henríquez Ureña, Santo Domingo, DOM; 3 Medicine, Weill Cornell Medical College - Qatar, Doha, QAT

**Keywords:** fishhook, neck trauma, pneumomediastinum, surgical emphysema, unilateral vocal cord paresis

## Abstract

Surgical emphysema can be seen with tracheal or esophageal injuries. Physicians should consider the possibility of subtle esophageal or tracheal injuries and investigate accordingly.

A 33-year-old previously healthy fisherman accidentally struck his neck with a fishhook while fishing and subsequently fell, sustaining combined blunt and penetrating trauma to the neck. In addition to a thorough medical examination, the patient underwent a chest X-ray, contrast-enhanced computed tomography (CECT), a barium swallow, and laryngobronchoscopy.

The findings included extensive surgical emphysema, vocal cord palsy, and pneumomediastinum with no visible injuries to the aerodigestive tract. The patient was monitored and conservatively treated. The surgical emphysema subsided within the next four to five days, and the patient was discharged on the seventh day without any complications.

A thorough clinical assessment, along with imaging, is essential to exclude life-threatening neck injuries. In hemodynamically stable patients with surgical emphysema and no radiological evidence of aerodigestive tract injury, conservative management with close monitoring can be an appropriate option.

## Introduction

Cervical surgical emphysema occurs when air is present beneath the fascia or soft tissue, leading to neck swelling [1]. The most common causes are trauma to the aerodigestive tract. The emphysema can spread to other body parts along the facial planes, including the chest wall, mediastinum, face, etc. [2]. The common causes of surgical emphysema and pneumomediastinum are trauma to the thoracoabdominal region that causes perforations of the trachea, bronchial tube, lung, esophagus, or a hollow abdominal viscus. Extensive cervical surgical emphysema due to minor neck trauma is rare, and air rarely descends from the cervical structures into the mediastinum. We present a case of extensive surgical emphysema of the neck with vocal cord palsy and pneumomediastinum due to blunt trauma and fishhook injury to the neck without aerodigestive tract injury. To the best of our knowledge, no such case has been reported in the literature.

## Case presentation

**Figure 1:**
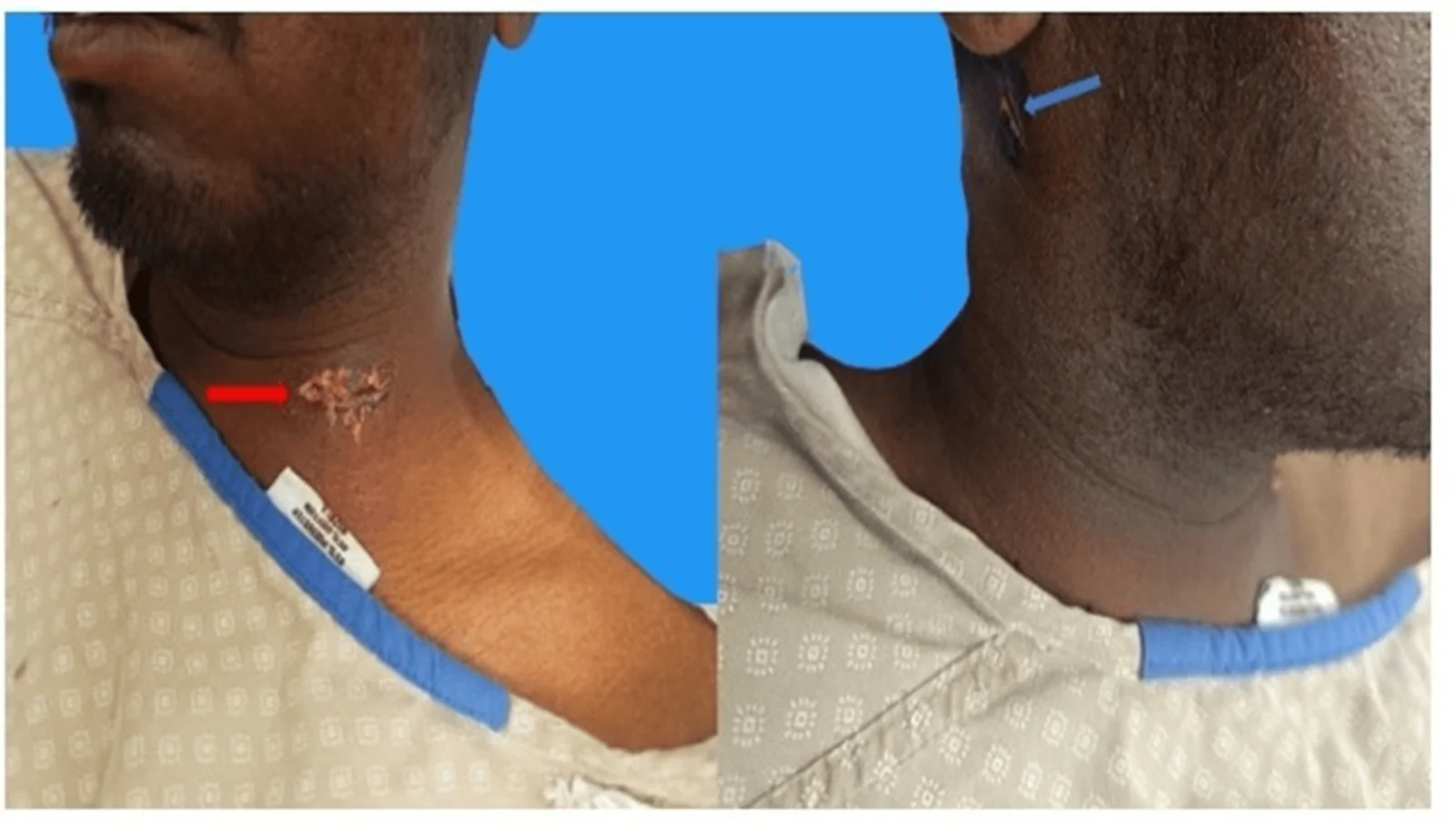
Fishhook injury site (red arrow) on the left side and another wound (blue arrow) on the right side of the neck

A 33-year-old previously healthy fisherman accidentally hit his neck with a fishhook while he was fishing. He was initially managed at another hospital and then referred to our hospital with an alleged history of neck trauma. At the same time, he threw a bunch of fishhooks into the water, and one hook accidentally hit the left side of his neck. Following this, he fell to the floor of the boat and was struck by an iron rod on the right side of his neck. The patient complained of mild neck pain and new hoarseness of voice. On examination, there were two wounds on the left and right sides of the neck (Figure 1).

**Figure 2:**
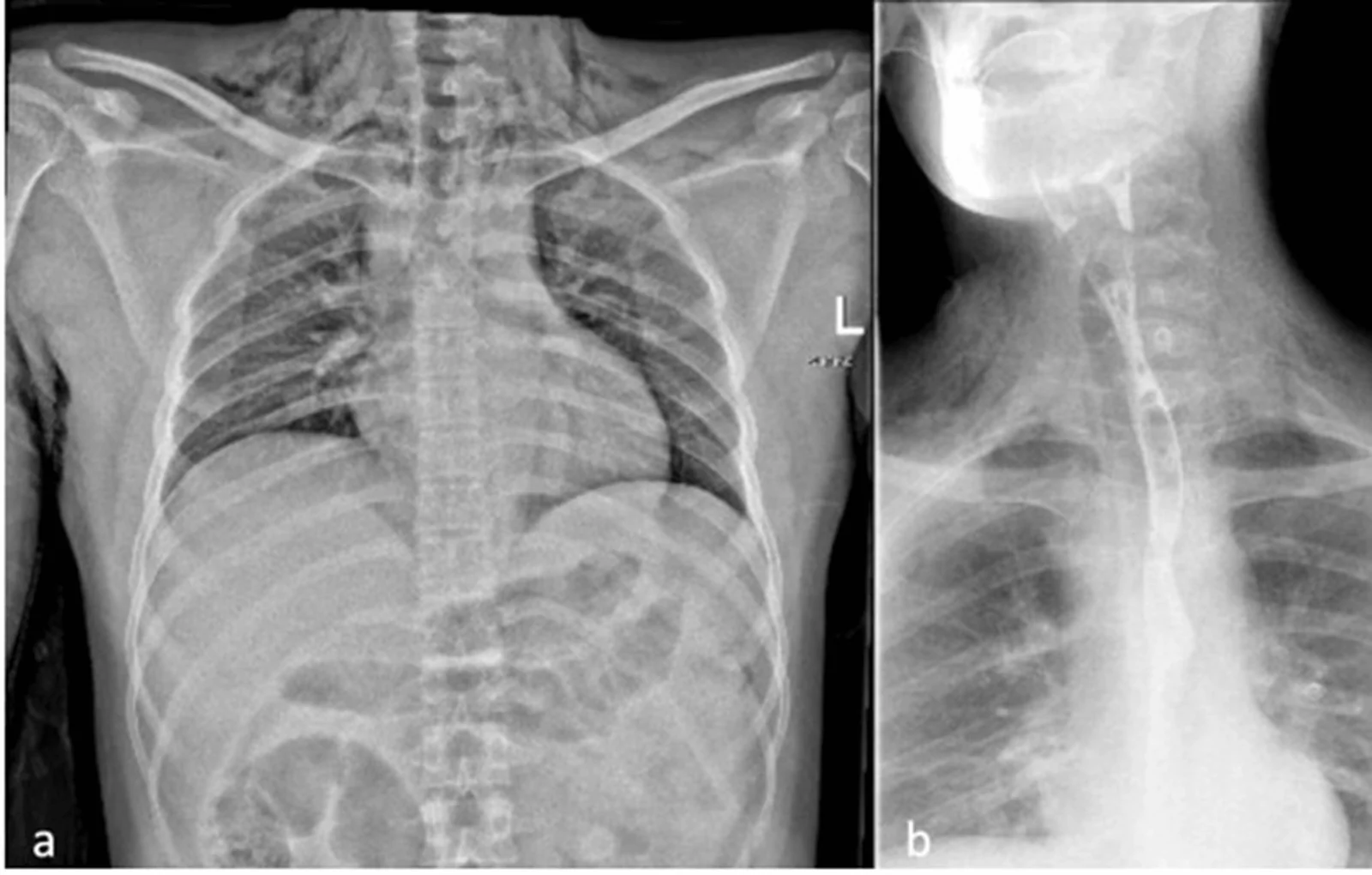
(a) Chest X-Ray: Diffuse emphysematous changes in the superficial and deep muscle planes in the neck. (b) Barium swallow: smooth outline with no filling defects and no contrast leak from the esophagus.

The left-side neck wound was in zone II and about 2.5 cm in size, without a platysma muscle breach. Another cut wound approximately 2 cm was also in zone II, just below the ear and posterior to the sternocleidomastoid. On palpation, a crackling sound was felt over the anterior and lateral sides of the neck. The plain chest X-ray showed surgical emphysema in the superficial and deep muscle planes of the neck bilaterally (Figure 2a). A barium swallow was performed to rule out esophageal injury. No obvious evidence of contrast leak from the esophagus was noted (Figure 2b). The esophagus demonstrated a smooth outline with no evidence of filling defects or abnormal external compressions.

**Figure 3:**
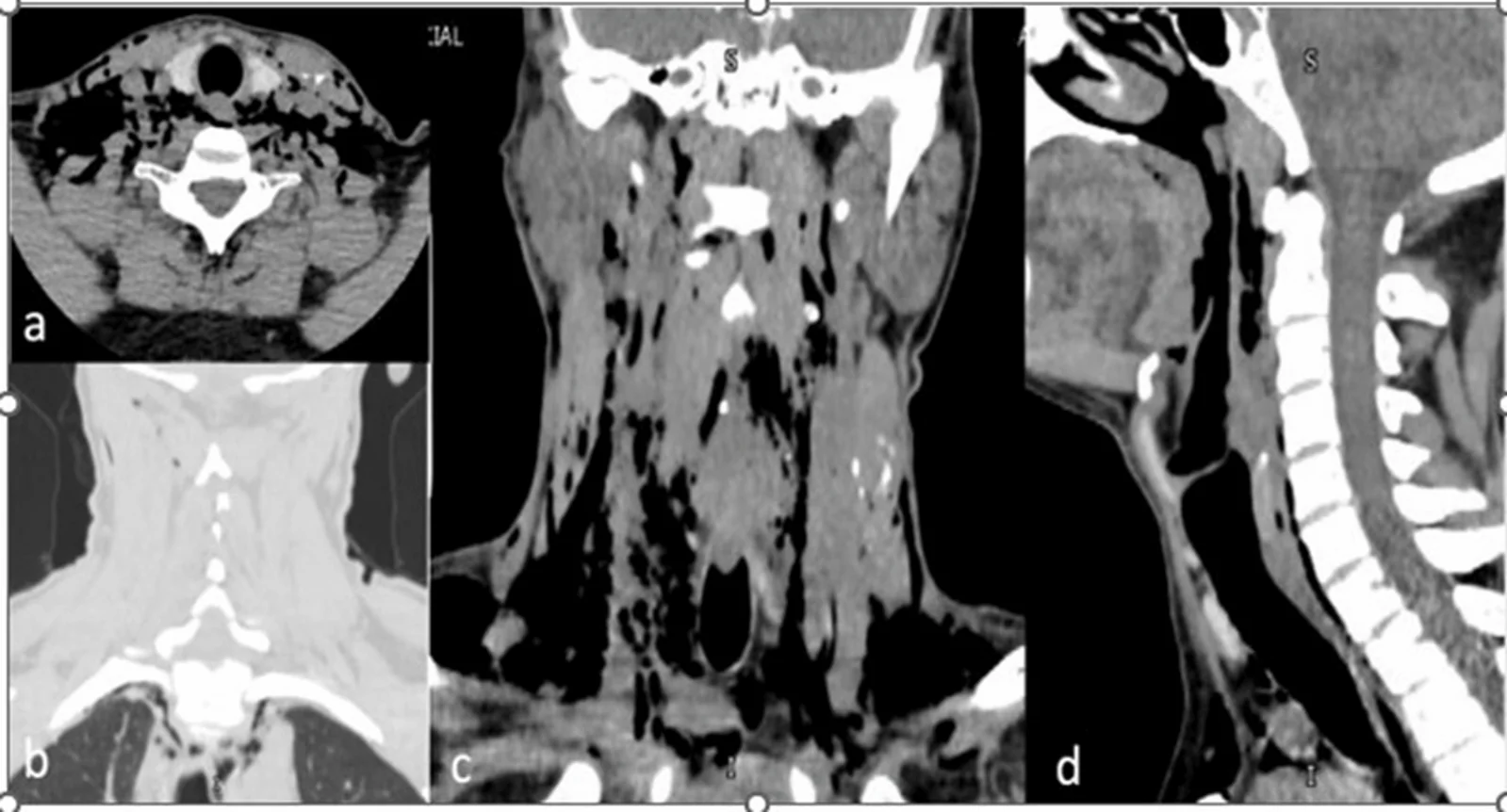
Axial (a) and coronal (c) views of computed tomography showing extensive surgical emphysema with air dissecting soft tissue planes of the neck. (b) Coronal view showing pneumomediastinum. (d) Sagittal view showing intact airways

Contrast-enhanced computed tomography (CECT) revealed extensive surgical emphysema, with air dissecting the soft tissue planes of the neck and pneumomediastinum (Figure 3a-d). No injuries were noted in the aerodigestive tract (Figure 3d).

**Figure 4:**
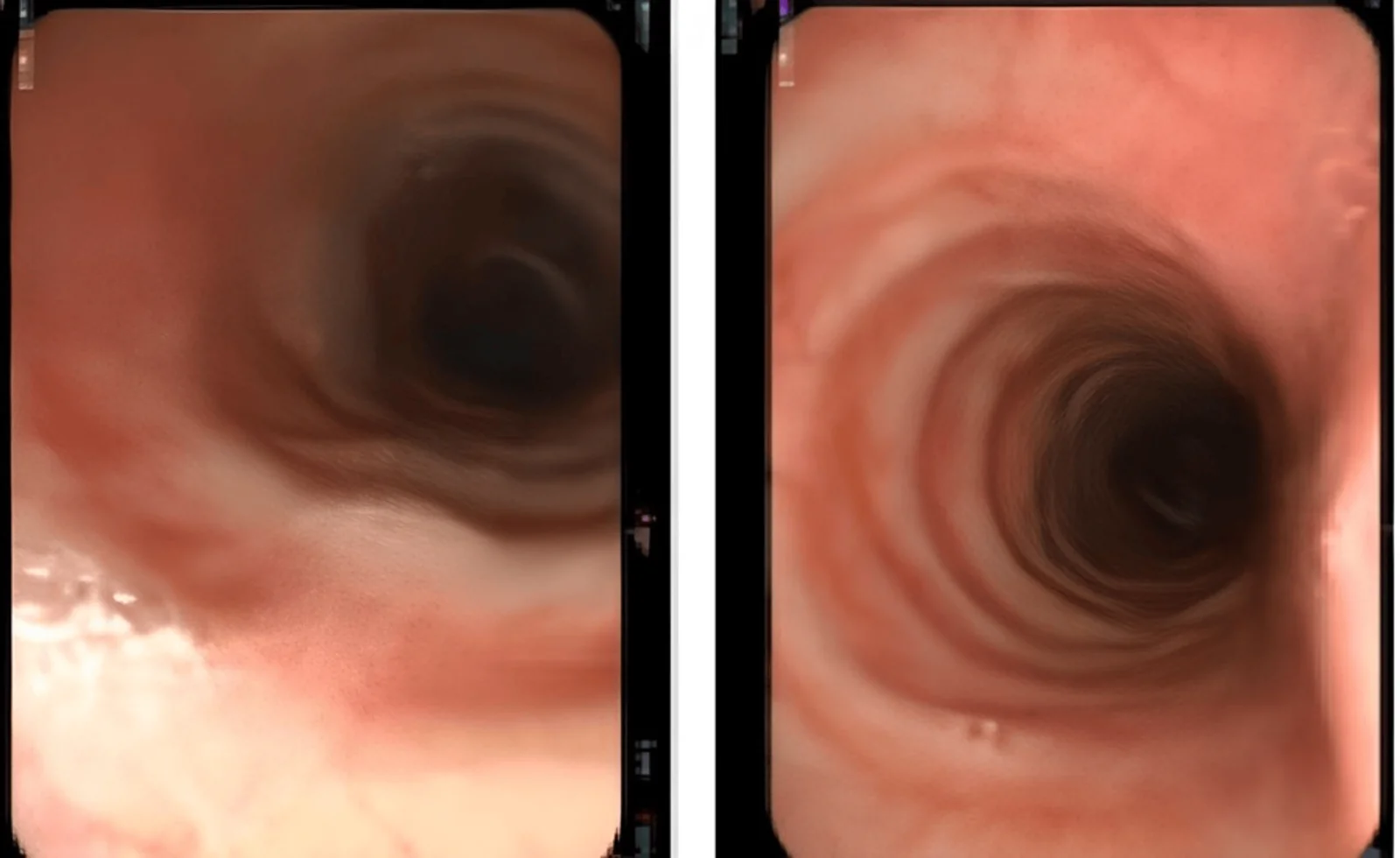
Bronchoscopy shows normal tracheal and bronchial tree

**Figure 5:**
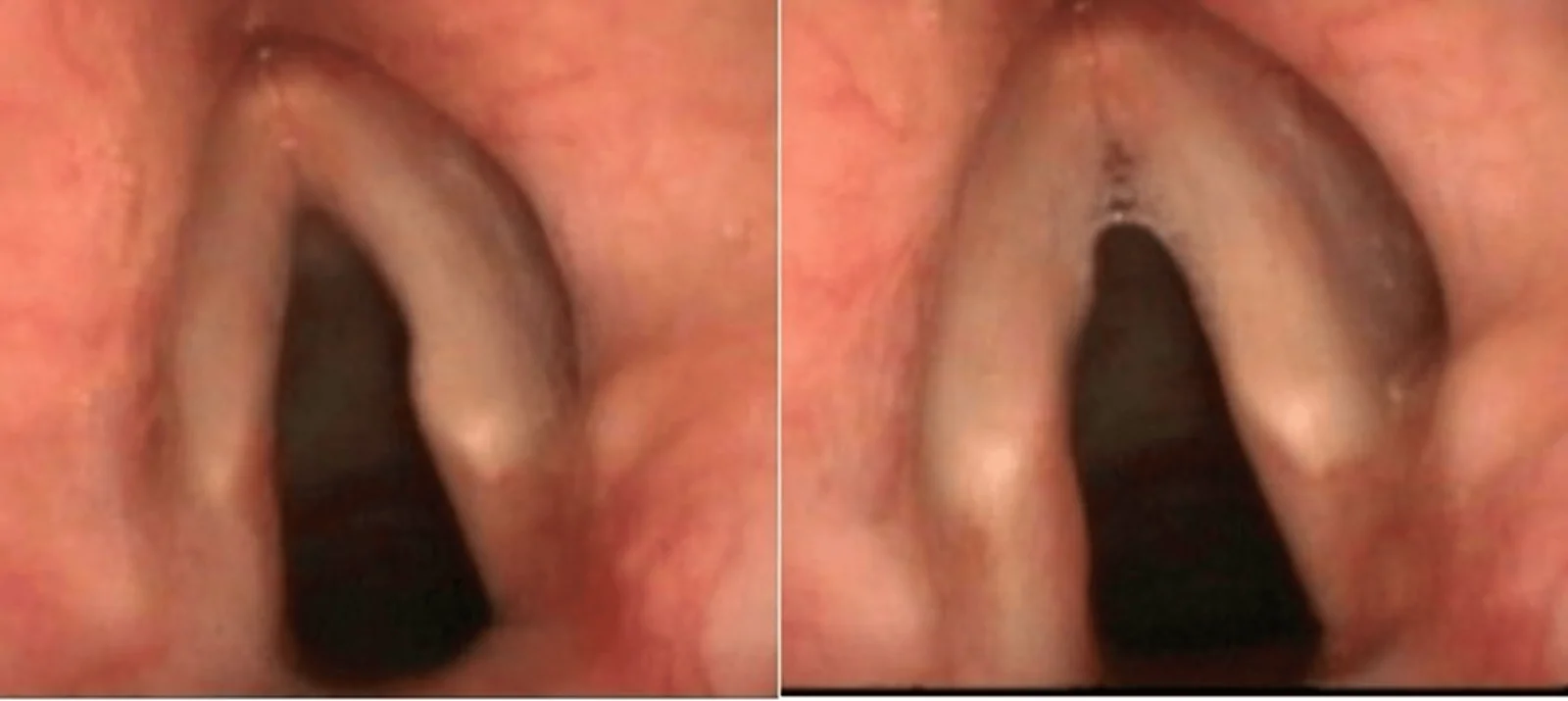
Laryngoscopy shows the right vocal cord is not moving,while the left cord is moving normally.

Laryngo-bronchoscopy revealed that the right vocal cord was not moving, and no injuries were observed in the nasopharyngeal mucosa, trachea, and both right and left bronchial tree (Figures 4, 5).

The patient was admitted under trauma surgery service and kept under close monitoring. Antibiotics and analgesics were administered according to the hospital protocol. The patient was maintained on a nothing per os (NPO) regimen for the first 24 hours. Later, he was allowed to resume a regular oral diet. The surgical emphysema subsided within the next four to five days, and the patient was discharged on the seventh day without any complications apart from mild hoarseness of voice.

## Discussion

To our knowledge, a constellation of vocal cord palsy, pneumomediastinum, and surgical emphysema following fishhook injury has not been reported in the literature. Neck pain with swelling must be differentiated from expanding hematoma and major tracheobronchial injuries. Patients can be selected cautiously and managed nonoperatively when investigations do not suggest injuries to the aerodigestive tract.

Trauma to the neck can be due to blunt or penetrating injury [3]. The neck contains vital neural, vascular, and aerodigestive structures, and injury to the neck can be life-threatening. Subcutaneous emphysema of the neck can occur after trauma to the aerodigestive tract in the neck, face, or chest. Apart from trauma, there are also other causes of surgical emphysema in the neck [4-7]. In the present report, the mechanism of injury involved penetrating trauma from a fishhook and an iron rod, as well as blunt trauma resulting from a fall.

The clinical features depend on the extent and degree of the surgical emphysema and associated injuries. Typical signs and symptoms of surgical emphysema include neck swelling, crepitus on palpation, neck pain, stiffness, dyspnea, and dysphagia [2,8]. Rarely, this condition can be serious and life-threatening if it affects deeper tissues and the thoracic outlet. Lung expansion is restricted if airway pressure is high; tension pneumothorax and respiratory failure can occur [9].

To identify the injury site, patients with any of the above signs and symptoms must undergo a thorough clinical examination and investigations, including CECT, fiberoptic bronchoscopy, and barium study. Karadağ et al. [2] also reported a case of penetrating neck trauma with surgical emphysema without injury in the trachea or esophagus. The two possible causes of surgical emphysema in the present case could be that air traveled directly from the open injury in the neck to the superficial and deep neck spaces and mediastinum, or a subtle injury to the trachea or esophagus, which was not picked up by the investigations or the Macklin effect [10] where sudden trauma causes the alveolar rupture with air dissecting centrally along the bronchovascular sheaths into the mediastinum and may track superiorly into the fascial planes of the neck. Laryngeal nerve injury is usually a complication of neck surgery, particularly thyroid and parathyroid gland surgeries [11] but is rarely seen in neck trauma. The right-sided vocal cord palsy in our case was most likely due to blunt trauma sustained when the patient fell after the fishhook struck his neck, resulting in neuropraxia.

## Conclusions

Surgical emphysema and pneumomediastinum following neck trauma, in the absence of aerodigestive tract injury, may result from an open neck injury or the Macklin effect following blunt trauma. In hemodynamically stable patients with no radiological evidence of aerodigestive tract injury, conservative management with close monitoring can be a safe and appropriate approach. Thorough clinical evaluation with appropriate imaging is essential to rule out life-threatening injuries.

## References

[1] Vargo RJ, Potluri A, Yeung AY, Aldojain A, Bilodeau EA (2016). Cervicofacial subcutaneous emphysema: a clinical case and review of the literature. Gen Dent.

[2] Karadağ D, Doner E, Adapınar B (2011). Thyroid emphysema following penetrating neck trauma. Balkan Med.

[3] Dar PM, Boddeda J, Kaur S (2022). Varied presentations, magnitude, and outcome of traumatic neck injuries at a level I trauma center. Emerg Crit Care Med.

[4] Iqbal M, Ikram M, Raza F, Banday N (2005). Surgical emphysema in the neck as a result of a dental procedure. Ear Nose Throat J.

[5] McKenzie WS, Rosenberg M (2009). Iatrogenic subcutaneous emphysema of dental and surgical origin: a literature review. J Oral Maxillofac Surg.

[6] Wilson M, Browne JD, Martin T, Geer C (2012). Case report: atypical presentation of jugular foramen mass. Am J Otolaryngol.

[7] Thompson C, Gohil R (2017). Pneumatic dental extractions: an unusual cause of extensive cervical surgical emphysema. BMJ Case Rep.

[8] Livingston DH (2023). Survivorship. J Trauma Acute Care Surg.

[9] Beck PL, Heitman SJ, Mody CH (2002). Simple construction of a subcutaneous catheter for treatment of severe subcutaneous emphysema. Chest.

[10] Dar P, Boddeda J (2026). Unmasking the Macklin effect: a rare case of coexisting pneumothorax and pneumomediastinum triggered by the Macklin effect. J Inj Acute Care.

[11] Kwak M, Mehaffey JH, Hawkins RB (2019). Bariatric surgery is independently associated with a decrease in the development of colorectal lesions. Surgery.

